# Cationic Peptide-Modified Gold Nanostars as Efficient Delivery Platform for RNA Interference Antitumor Therapy

**DOI:** 10.3390/polym13213764

**Published:** 2021-10-30

**Authors:** Si Chen, Jiguang Li, Xiaoyu Ma, Fan Liu, Guoping Yan

**Affiliations:** 1 Hubei Key Laboratory of Plasma Chemistry and Advanced Materials, School of Material Science and Engineering, Wuhan Institute of Technology, Wuhan 430205, China; ljg2434783404@163.com (J.L.); mxy18734564886@163.com (X.M.); fan.liu@wit.edu.cn (F.L.); 2 Key Laboratory of Biomedical Polymers of Ministry of Education, College of Chemistry and Molecular Sciences, Wuhan University, Wuhan 430072, China

**Keywords:** gene therapy, peptide, siRNA carrier, gold nanostars, tumor targeting

## Abstract

siRNA interference therapy can silence tumor cell target genes and specifically regulate tumor cell behavior and function, which is an effective antitumor therapy. However, in somatic circulation, naked siRNAs are not only susceptible to degrade, but it is also difficult to realize the tumor cells’ internalization. Therefore, novel siRNA delivery vectors that could promote efficacy need to be developed urgently. Here, we designed high-surface gold nanostars (GNS-P) which are decorated with cationic tumor-targeting peptide as an efficient and functional siRNA delivery nanoplatform for tumor therapy. The positively charged amino acid sequence and huge surface area enabled the vector to load a large amount of siRNA, while the tumor-targeting peptide sequence and nano size enabled it to rapidly and precisely target the tumor regions for fast and effective siRNA delivery. This tumor-targeting nanoplatform, GNS-P, displayed good biocompatibility, low toxicity and an extraordinary tumor accumulation capability.

## 1. Introduction

In the process of fighting against COVID-19, messenger RNA (mRNA) vaccines play an important and significant role in inhibiting virus transmission. The characteristics of high safety, high protection rates and easy production have reactivated the interest of researchers in RNA [1,2]. In fact, vaccines are only one of the emerging applications of RNA, while RNA interference (RNAi) therapies based on gene silencing mechanisms have been used in the treatment of refractory diseases such as antitumor therapy [3,4]. As a typical representative of RNAi, small interfering RNA (siRNA) is a type of short double-stranded RNA which enables sequence-specific gene silencing of complementary mRNA, induces mRNA degradation and inhibits the production of target proteins [5,6]. By utilizing siRNA to induce expression silencing of target genes and to regulate transcriptional processes of control corresponding to proteins (such as Bcl-2 family proteins that regulate programmed cell death or apoptosis), RNAi therapy could achieve the inhibition of tumor progression and improve antitumor efficiency [7]. Moreover, the ability of such nucleic acid-based treatments could evolve dynamically in response to mutations in the targeted tumor site, which greatly improves the adaptability of tumor therapy [8]. In the meantime, the functions of siRNA are prior to protein expression, enabling precise control over the function and behavior of tumor cells upstream, which is difficult to achieve with many conventional therapies [9,10]. 

However, siRNA is essentially a ribonucleic acid and generally possesses high molecular weight (M.W. ≈ 11−14 kDa), with an overall negatively charged phosphate backbone [11]. These characteristics make it difficult for the naked siRNA chain to be absorbed by tumor cells with a negative surface charge and to pass through the outer bilayer lipid membrane of tumor cells, which greatly limits the function of siRNA [12]. In addition, the naked siRNA faces many challenges such as a rapid metabolism, the degradation of RNA enzymes and poor homing of target cells during systemic circulation [13,14]. In view of this, in order to maximize the efficiency of siRNA gene silencing, it is urgent to develop a novel delivery platform with precise targeting and long circulation protection functions to help siRNA identify tumor cells accurately and enter tumor cells completely [15,16]. Peptides are a class of biomolecules polymerized by natural amino acids, whose specific sequence composition endows them with corresponding biological activities and functions [17,18,19]. Customized cationic peptide-modified nanoparticles can achieve the targeted delivery of siRNA and are regarded as promising siRNA delivery platforms [20,21].

**Figure 1:**
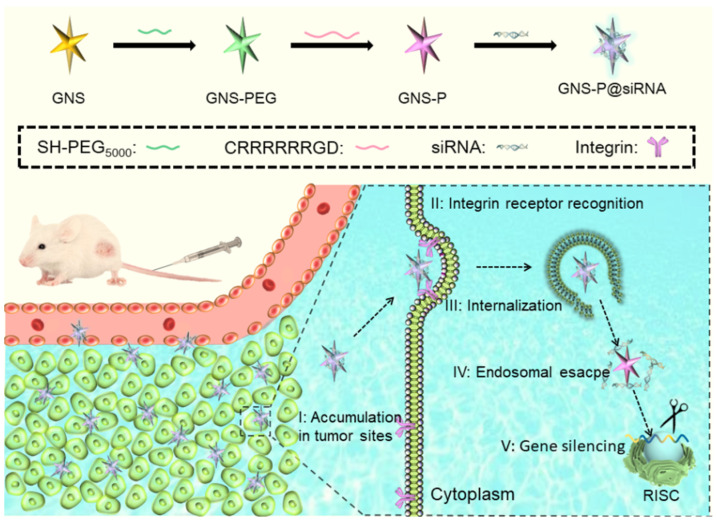
Schematic diagram of the antitumor process of GNS-P as the siRNA delivery platform.

Here, we designed and constructed cationic tumor-targeting peptide-modified gold nanostars (GNS-P) as an efficient delivery platform for RNAi antitumor therapy to overcome the therapeutic inefficiency of naked siRNA in vivo. Compared with conventional nanocarriers, the anisotropic tentacles of gold nanostars (GNS) provided a higher specific surface area and more modification sites on the surface of nanoparticles, while the nanoparticles facilitate the enrichment of tumor regions through trans-endothelial pathways [22,23]. Therefore, we chose GNS as the template material and modified PEG on GNS surfaces to obtain the nanoparticle GNS-PEG with higher stability [24]. As shown in Figure 1, we designed and synthesized the cationic tumor-targeting peptide CRRRRRRGD and anchored the peptide on GNS-PEG with a gold-sulfur bond to obtain the functional siRNA delivery nanoplatform GNS-P [25]. The arginine-rich fragment of cationic tumor-targeting peptide could provide a large positive charge on the surface of GNS-P, enabling it to transport siRNA through electrostatic adsorption amply. Moreover, the RGD integrin receptor sequence in peptide can recognize the α_v_β_3_ protein, facilitating the capture and endocytosis of the modified GNS-P by tumor cells [26,27]. In vitro experiments demonstrated that nanoplatform GNS-P could deliver siRNA to tumor cells effectively while in vivo experiments demonstrated GNS-P could achieve satisfactory tumor targeting enrichment. This nanoplatform provides a simple and effective strategy to customize siRNA delivery vectors for tumor therapy.

## 2. Materials and Methods

### 2.1. Materials

Rink Amide-AM resin (0.8 mmol/g), L-amino acids protected by N-Fluorenyl-9-methoxycarbonyl (Fmoc) (Fmoc-Cys(Trt)-OH, Fmoc-Arg(Pbf)-OH, Fmoc-Gly-OH and Fmoc-Asp(OtBu)-OH), 1-hydroxybenzotriazole (HOBt) and O-benzotriazole-N,N,N′,N′-tetramethyl-uronium-hexafluoro-phosphate (HBTU) were provided by GL Biochem. Ltd. (Shanghai, China). Diisopropylethylamine (DIEA), N,N-dimethylformamide (DMF), piperidine, trifluoroacetic acid (TFA), methanol, dichloromethane (DCM), thioanisole, ethanedithiol (EDT) and anhydrous ether were obtained from Shanghai Chemical Reagent Co. (Shanghai, China). The 4-(2-hydroxyethyl)-1-piperazineethane-sulfonic acid (HEPES) buffers, hydrogen tetrachloroaurate (III) trihydrate (HAuCl_4_) and Lipofectamine 2000 (Lipo2000) were provided by Sigma-Aldrich Co. LLC.

### 2.2. Synthesis of Tumor-Targeting Peptide

**Figure 2:**
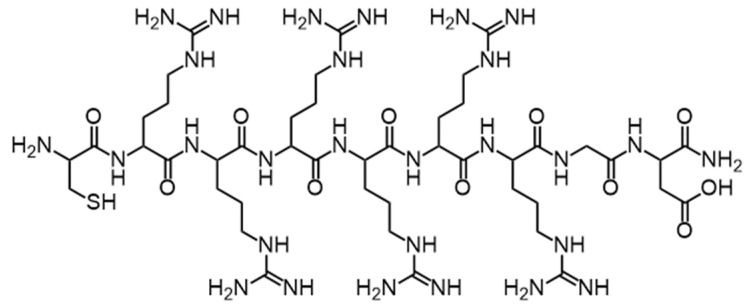
Schematic structure of the cationic peptide CRRRRRRGD.

Tumor-targeting peptide CRRRRRRGD (Cys-Arg-Arg-Arg-Arg-Arg-Arg-Gly-Asp, the sequence of peptide as shown in Figure 2) was synthesized on Rink Amide-AM resin using a standard Fmoc-based solid-phase synthesis technique [28,29]. Afterwards, tumor-targeting peptide was cleaved from the resin by cleavage cocktail. After the cleavage mixture is collected, it is further concentrated and precipitated in ether. The collected precipitate was lyophilized before further use. The purity of peptide was assured to be above 90% by analytical high performance liquid chromatography (HPLC). The molecular weight of peptide was detected by electrospray ionization mass spectrometry (ESI-MS).

### 2.3. Synthesis of GNS and GNS-PEG

GNS was synthesized in a HEPES buffer by reducing HAuCl_4_ [30,31]. Briefly, GNS was synthesized by adding 1 mL of 40 mM HAuCl_4_ to 100 mL of 100 mM HEPES buffer solution. Then, the solution was adjusted to pH 7.4 and left to stand at room temperature for 1 h until the color changed to blue green. Afterwards, the product collected by centrifugalization was GNS. The appearances of GNS were visually observed by Transmission Electron Microscopy (TEM) and Scanning Electron Miscopy (SEM).

Then, GNS-PEG was synthesized by mixing GNS and HS-PEG_5000_-OCH_3_ at a *w*/*w* ratio of 5:1 and stirring for 12 h. 

### 2.4. Agarose Gel Retardation Assay

The ability of loading siRNA to form a stable complex was a prerequisite of siRNA delivery vectors. Additionally, the grafting degree of peptides affected the ability of GNS-P to compress siRNA directly. In order to find the optimal grafting degree of peptides, the siRNA condensing capabilities of GNS-P were examined at different grafting degrees of peptide by agarose gel retardation assay. Naked siRNA was used as a negative control. GNS-P (w_Peptide_/w_PEG_ ranging from 0.5 to 6), Lipo2000 or GNS-PEG were mixed with 0.1 μg siRNA (w_sample_/w_siRNA_ = 10:1), labeled by bromophenol blue and diluted to 10 μL with PBS (10 mM). The mixing solutions were incubated at 37 °C for 0.5 h and electrophoresed on the agarose gel containing GelRedTM (*w*/*v*: 0.7%) in a Tris-acetate (TAE) running buffer at 80 V for 1 h [32]. 

### 2.5. Synthesis of GNS-P and GNS-P@siRNA

According to the result of the agarose gel retardation assay, GNS-P could condense siRNA at w_Peptide_/w_PEG_ = 6. Then, GNS-P was obtained by mixing GNS-PEG and peptide at a w_Peptide_/w_PEG_ ratio of 6 and stirring for 12 h. 

GNS-P@siRNA was obtained by adding 1 μg siRNA (w_GNS-P_/w_siRNA_ = 10:1) into appropriate GNS-P solutions (1 mg/mL) and incubated at 37 °C for 0.5 h.

### 2.6. Characterization of GNS, GNS-PEG and GNS-P

The particle size and the zeta potential of GNS, GNS-PEG and GNS-P were detected by Nano-ZS ZEN3600 (Malvern Instruments).

The absorbance spectra of GNS, GNS-PEG and GNS-P were measured at 25 °C by using UV-Vis spectrophotometer (Lambda Bio40, Perkin-Elmer, Waltham, MA, USA) [33].

### 2.7. Intracellular Uptake and Endosomal Escape Behavior

Intracellular uptake behaviors of GNS-P@siRNA in murine pancreatic adenocarcinoma (Panc02) cells were observed by confocal laser scanning microscope (CLSM) (C1-Si, Nikon). A total of 1 μg of Cy5 labeled siRNA (siRNA-Cy5) was added into GNS-P solution (w_GNS-P_/w_siRNA_ = 10:1) and incubated at 37 °C for 0.5 h to prepare GNS-P@siRNA-Cy5. Afterwards, the samples were supplemented by a medium with 10% FBS to 1 mL and incubated with Panc02 cells for 4 h. Finally, after the cell nuclei was stained with Hoechst 33342, the fluorescence images of samples were acquired by CLSM. Naked siRNA-Cy5 was used as a control [34] and the quantitative analysis of siRNA levels after cellular internalization was further tested by flow cytometry.

The endosomal escape behavior of GNS-P@siRNA in Panc02 cells was observed by CLSM, Lipo2000@siRNA was used as a control. siRNA was labeled by Cy5. GNS-P@siRNA-Cy5 and Lipo2000@siRNA-Cy5 (wsample/wsiRNA = 10:1) were incubated with cells for 4 h, 8 h or 12 h. Finally, cell endosomes were labeled with LysoTracker Green and cell nuclei were stained with Hoechst 33342.

The fluorescence of cell nuclei was obtained by using the 408 nm line of laser, the cell endosome was obtained by using the 488 nm line of laser and the siRNA-Cy5 was obtained by using the 650 nm line of laser.

### 2.8. Cytotoxicity Assay In Vitro

The cytotoxicity of GNS, GNS-PEG and GNS-P in Panc02 cells was evaluated via MTT assay. The cytotoxicity of Lipo2000 was used as a control. After the cells were seeded in a 96-well plate for 24 h, the samples at different concentrations were added into the plate and incubated with the cells for 48 h. Afterwards, 20 μL MTT solution was added to the cells for 4 h of incubation. Finally, the medium was replaced with 200 μL DMSO. The absorbance at 570 nm was tested by microplate reader (Model 550, Bio-Rad). The survival rate was defined as follows: survival rate (%) = (OD570_samples_ − OD570_blank_)/(OD570_control_ − OD570_blank_) × 100. The OD570_control_ was detected in the absence of samples while OD570_samples_ were tested in the presence of samples [35].

### 2.9. Gene Silencing Analysis

After Panc02 cells were seeded in a 6-well plate for 24 h, GNS@siRNA, GNS-PEG@siRNA, GNS-P@siRNA or Lipo2000@siRNA (the dose of siRNA was 1μg, w_sample_/w_siRNA_ = 10:1) in 2 mL of medium were added to the plate with Panc02 cells for 4 h of incubation. Then, the samples’ medium was replaced with fresh medium containing 10% FBS. After 48 h of incubation, the Bcl-2 expression of samples were tested by western blotting analysis and enzyme-linked immunosorbent assay (ELISA) [36].

### 2.10. In Vivo Biodistribution Study

The female C57/BL6 mice (20 g) employed in this study were purchased from the Hubei Provincial Center for Disease Control and Prevention. A subcutaneous tumor model was used for the study. The tumor-bearing mice were injected subcutaneously with Panc02 cells in the right side of their backs. After the tumors’ volume reached 150 mm^3^, the mice were I.V. injected with GNS-P@siRNA-Cy5 or Lipo2000@siRNA-Cy5 (the dose was 10mg/kg). After injection for 0 h or 12 h, the distribution of samples was analyzed with the Spectrum Preclinical In Vivo Imaging System (IVIS) [37].

## 3. Results and Discussion

Specific anti-apoptotic mechanisms exist in tumor cells to avoid the onset of apoptosis, thus rendering tumor cells immortal. The Bcl-2 family of proteins is a series of proteins that regulates programmed cell death or apoptosis and all members contain at least one of the four Bcl-2 homology domains, some of which (Bcl-2, Bcl-XL and Mcl-1) are anti-apoptotic, while others (Bax, Bak and Bok) are pro-apoptotic. Among them, the Bcl-2 protein regulates cell death by controlling mitochondrial membrane permeability and inhibits caspase activity by blocking the release of cytochrome C from mitochondria and/or binding to the apoptosis activating factor (APAF-1). Therefore, the gene-silencing targeting Bcl-2 protein is an effective gene therapy strategy to promote apoptosis in tumor cells.

In this research, in order to use the corresponding siRNA of the Bcl-2 protein-coding gene to achieve precise targeted silencing of the Bcl-2 gene, we designed and constructed a cationic tumor-targeting peptide-modified gold nanostar (GNS-P) as an efficient delivery platform for RNAi antitumor therapy in vivo (Figure 1). GNS with high specific surface area, low toxicity and controlled synthesis was selected as the carrier template. Additionally, GNS-PEG, which modified PEG on GNS, could improve circulation time and biocompatibility in vivo. Further, we synthesized the cationic tumor-targeting peptide CRRRRRRGD and decorated the surface of GNS-PEG to obtain the nanoplatform GNS-P. The cationic tumor-targeting peptide CRRRRRRGD provides an extremely strong positive charge of GNS-P, enabling the siRNA to bind stably on the system by electrostatic adsorption to form GNS-P@siRNA. The outermost RGD integrin receptor sequence, which can recognize integrins on the tumor cell surface for tumor-specific targeting. After GNS-P@siRNA enters the body circulation, it is rapidly localized within the tumor region by trans-endothelial pathways and RGD-integrin recognition. The overall positive charge promotes the transcytosis of GNS-P@siRNA by tumor cells into the tumor cell lysosomes and the proton sponge effect of the arginine-rich peptide in the acidic lysosomal environment prompts the platform to swell and release the siRNA into the cytoplasm, acting on the target mRNA and silencing the target gene.

### 3.1. Synthesis and Characterization of Cationic Tumor-Targeting Peptides

**Figure 3:**
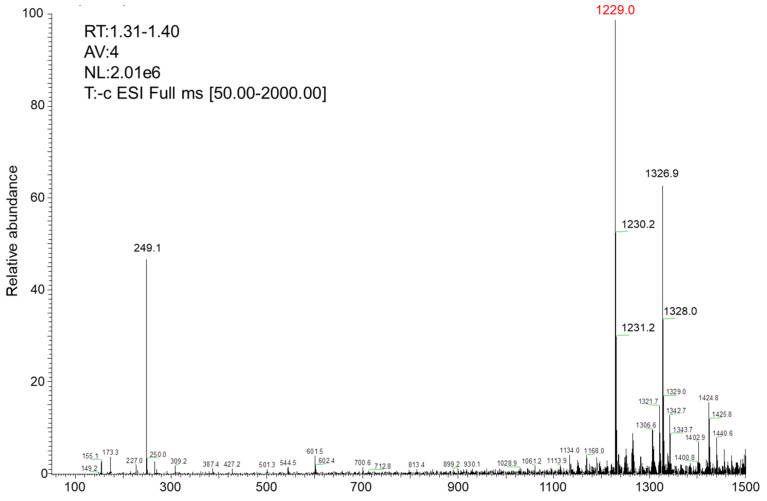
ESI-MS of the cationic peptide CRRRRRRGD.

First, the cationic tumor-targeting peptide with the sequence CRRRRRRRGD (Figure 2) was synthesized by using standard solid-phase synthesis methods. The molecular weight of the target peptide should be calculated at 1228.69. We determined the molecular weight of the peptides using ESI-MS and the results were as expected (Table 1, Figure 3). 

### 3.2. Construction and Characterization of GNS, GNS-PEG and GNS-P

**Figure 4:**
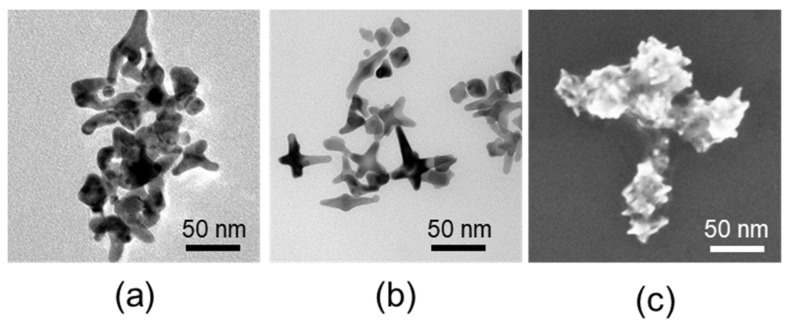
(**a**) TEM images of GNS; (**b**) TEM images of GNS-PEG; (**c**) SEM images of GNS.

Further, we have successfully synthesized nanoscale GNS by a reduction deposition method. GNS is a kind of star-like gold nanoscale particle formed by the reductive deposition of gold ions with multiple pointed arms with sharp corners. With an irregular tip structure and a large specific surface area, GNS have remarkable scattering abilities, a large molar extinction coefficient and a plasmon resonance effect in the infrared region. At the same time, due to the stable physicochemical properties and good biocompatibility, GNS is used as a contrast agent and nanocarrier in biomedical fields. Through the results of TEM and SEM in Figure 4, we can visualize that the unmodified GNS has a size of about 40 nm and is in the form of regular star-shaped particles. Compared with GNS, the dispersibility of GNS-PEG was significantly improved after the modification of PEG. The star-like shape of GNS, with its higher specific surface area, is beneficial to the high loading efficiency of siRNA, while the sharp corners of GNS could promote the enrichment of tumor regions based on trans-endothelial pathways and endocytosis by tumor cells.

**Figure 5:**
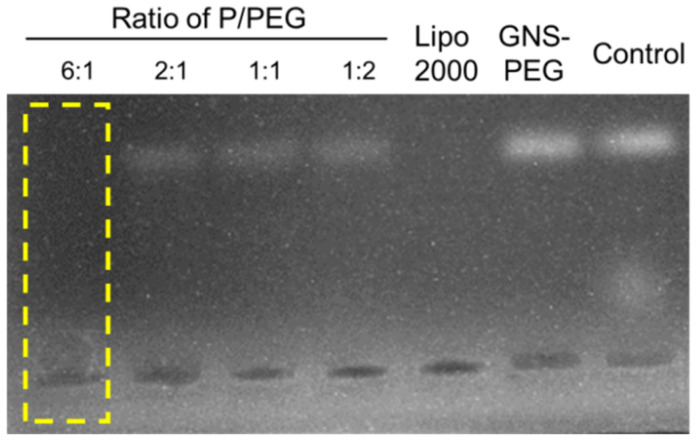
Optimization of the adsorption performance of GNS on siRNA analyzed by agarose gel electrophoresis.

After obtaining stable and uniformly distributed GNS, we attempted to coat their surfaces with PEG to enhance their long circulation time in vivo, reduce possible toxic side effects and stabilize their surfaces to avoid aggregation of subsequent cationic peptides. Subsequently, we explored the optimal P/PEG ratio by agarose gel electrophoresis at different P/PEG feeding ratios (w_Peptide_/w_PEG_ ranging from 0.5 to 6). Lipo2000 or GNS-PEG were mixed with siRNA (0.1μM) and labeled with bromophenol blue. The results in Figure 5 showed that the peptide-modified GNS-PEG had the best adsorption efficiency for siRNA under the condition of 6:1 peptide to PEG, and its siRNA loading efficiency could reach the level of the commercialized gene liposome vector Lipo2000.

**Figure 6:**
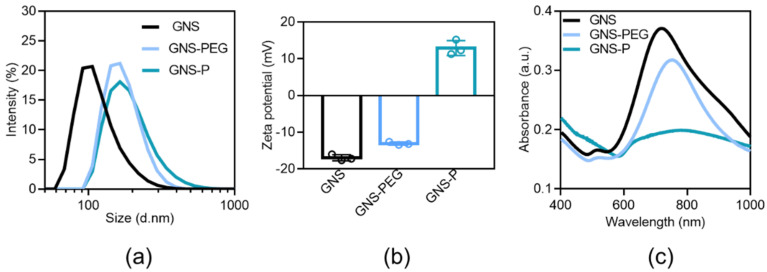
(**a**) Hydrated particle size distribution of GNS, GNS-PEG and GNS-P. (**b**) Surface potential of GNS, GNS-PEG and GNS-P. (**c**) UV Absorption Spectroscopy of GNS, GNS-PEG and GNS-P.

Further, we characterized the physicochemical properties of GNS, GNS-PEG and GNS-P. As shown in Figure 6a and Table 2, the hydrated particle size of GNS was about 100 nm, and after the modification of PEG, its hydrated particle size increased to about 170 nm due to the excellent hydrophilicity of PEG. The hydrated particle size increased slightly after further modification of peptide, indicating the successful modification of PEG with peptide on the surface of GNS. Correspondingly, the surface of GNS, prepared by reduction in HEPES buffer solution, was negatively charged around -18 mV. After PEG modification, the surface electrical properties changed to around -13 mV, while after further modification with cationic peptides, the surface charge had flipped to +14. The significant change on the surface electrical properties of nanoparticles shown in Figure 6b and Table 2 indicates that PEG and peptides were successfully modified on the surface of GNS, respectively. In the UV absorption spectra of this delivery system before and after the modification, the absorption peak at 720 nm did not change significantly (Figure 6c and Table 2), indicating that the PEGylation and the peptide modification process did not affect the structure of GNS themselves.

### 3.3. GNS-P Silences Target Genes at the Cellular Level

**Figure 7:**
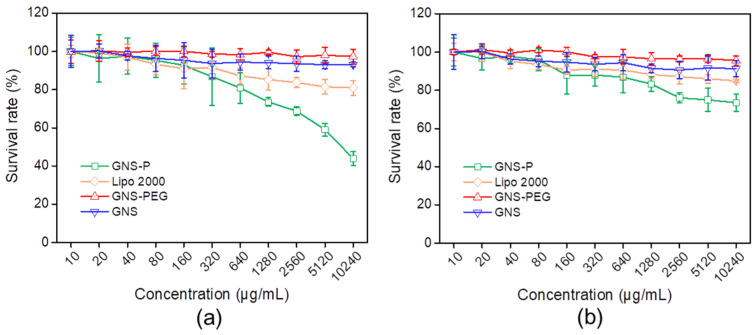
(**a**) Cytotoxicity of different concentrations of vectors on tumor cells Panc02. (**b**) Cytotoxicity of different concentrations of vectors on normal cells NIH3T3.

Since commonly used conventional gene vectors, including PEI, are usually more cytotoxic, they tend to kill both target and normal cells during gene delivery. Therefore, we further evaluated the cytotoxicity of samples on tumor cells as well as normal cells. As shown in Figure 7a, both GNS-P and Lipo2000 showed a decrease in cellular activity in tumor cells Panc02 with the higher concentration of the added vector. When the concentration increased to 640 ug/mL, more than 80% of tumor cells still survived, while for normal murine embryo fibroblast (NIH3T3) cells, the increase in vector concentration did not produce significant cytotoxicity (Figure 7b). With a further-increasing concentration, GNS-P showed stronger cytotoxicity in tumor cells Panc02 and a lesser effect on the survival of normal cells NIH3T3. Therefore, the above results indicate that GNS-P has negligible toxicity to normal cells and could be used as a safe siRNA delivery vector for tumor therapy.

**Figure 8:**
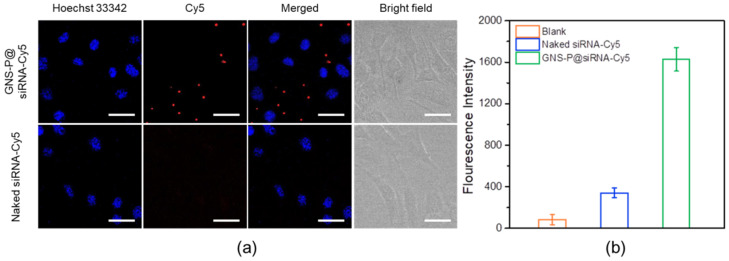
(**a**) Intracellular uptake of GNS-P@siRNA-Cy5 by Panc02 cells (scale bar: 100 μm); (**b**) Quantitative analysis of GNS-P@siRNA-Cy5 internalization by Panc02 cells.

In addition, the presence of cationic amino acids and RGD sequences endows GNS-P with the ability to recognize integrin receptors on the tumor cell membrane, with stronger electrostatic attraction and with the ability to cross the phospholipid bilayer of cell membranes. Based on this, we examined the process of GNS-P@siRNA recognition and internalization by tumor cells for endocytosis. GNS-P@siRNA-Cy5 was co-cultured with Panc02 cells for 4 h. Then, the nuclei of tumor cells were stained with Hoechst 33342 and the fluorescence images of samples were acquired by CLSM. Naked siRNA-Cy5 was used as a negative control. As shown in Figure 8a, after being co-cultured for 4 h, the red fluorescence of naked siRNA-Cy5 in the cells was almost negligible, while the fluorescence of GNS-P@siRNA-Cy5 could be observed in the cytoplasm of tumor cells significantly. Additionally, the quantitative analysis of GNS-P@siRNA-Cy5 recognition and endocytosis was further tested. As shown in Figure 8b, the fluorescence intensity of GNS-P@siRNA-Cy5 was much stronger than that of naked siRNA-Cy5. The data of the intracellular uptake demonstrated that GNS-P@siRNA could be internalized by tumor cells successfully.

**Figure 9:**
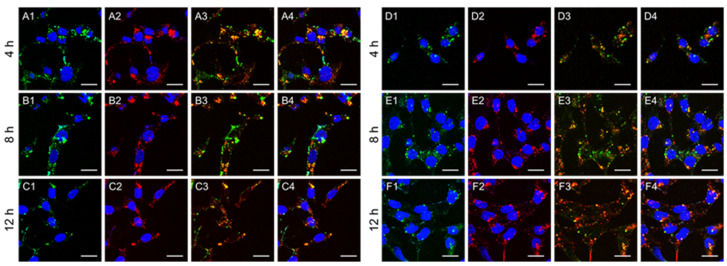
Endosomal escape of Lipo2000@siRNA-Cy5 (A–C) and GNS-P@siRNA-Cy5 (D–F) for 4 h, 8 h and 12 h by CLSM. The nucleus was stained with Hoechst 33342 (blue signal), siRNA was labeled by Cy5 (red signal) and the endosome was stained with LysoTracker Green (green signal). (A1–F1), overlay of endosome and nucleus; (A2–F2), overlay of siRNA and nucleus; (A3–F3), overlay of endosome and siRNA; (A4–F4), overlay of endosome, siRNA and nucleus. The scale bar was 20 μm.

After being internalized by tumor cells, GNS-P@siRNA would be trapped in the endosome. However, the environment of an endosome is not conducive to the biological activity of siRNA. To ensure that GNS-P@siRNA can escape from the endosome, we further verified the endosomal escape ability by CLSM. As shown in Figure 9, after 8 h of incubation, the isolated distribution of red fluorescence to siRNA-Cy5 from green fluorescence of endosome could be found in CLSM images, which implies that a part of the samples escaped from the endosome successfully.

**Figure 10:**
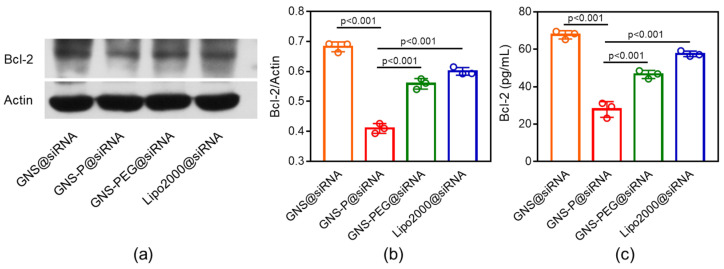
(**a**) The expression of Bcl-2 in Panc02 cells after incubation with different siRNA delivery platforms by western blotting analysis. (**b**) Quantitative analysis of Bcl-2 expression by western blotting analysis in Panc02 cells after incubation with different siRNA delivery platforms. (**c**) Quantitative analysis of Bcl-2 expression by ELISA in Panc02 cells after incubated with different siRNA delivery platforms. Significance between every two groups was calculated using one-way ANOVA. Data are mean ± S.D.

We further examined the silencing effect of internalized GNS-P on the tumor cell Panc02 anti-apoptotic gene Bcl-2. The corresponding siRNA of Bcl-2 was loaded on GNS-P (GNS-P@siRNA) and co-cultured with Panc02 cells for 4 h. GNS@siRNA, GNS-PEG@siRNA and Lipo2000@siRNA were used as a control. The expression levels of the Bcl-2 protein were examined by western blotting analysis and ELISA. As shown in Figure 10a,b, compared with other groups and even the commercial vector Lipo2000, GNS-P@siRNA showed a more effective gene silencing ability by western blotting analysis. Quantitative analysis of Bcl-2 expression by ELISA is shown in Figure 10c, the expression level of Bcl-2 is: GNS@siRNA > Lipo2000@siRNA > GNS-PEG@siRNA > GNS-P@siRNA, which is consistent with the trend of western blotting analysis and implies the excellent gene silencing effect of GNS-P@siRNA again.

### 3.4. In Vivo Biodistribution Study

**Figure 11:**
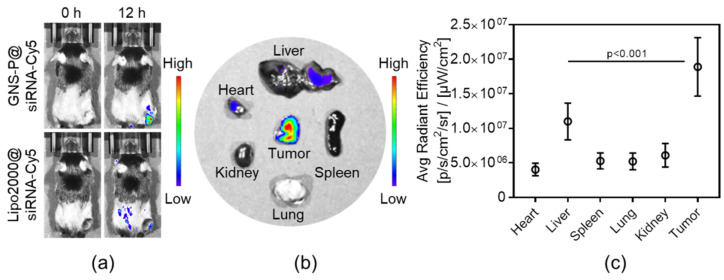
(**a**) Biodistribution of Panc02 tumor-bearing mice after I.V. injection of GNS-P@siRNA-Cy5 or Lipo2000@siRNA-Cy5 for 0 h or 12 h. (**b**) Biodistribution of tumors and major organs after I.V. injection of GNS-P@siRNA-Cy5 for 12 h. (**c**) Average radiant efficiency of tumors and major organs after I.V. injection of GNS-P@siRNA-Cy5 for 12 h. Significance between the liver group and the tumor group was calculated by one-way ANOVA. Data are mean ± S.D.

To further evaluate the targeting ability of GNS-P@siRNA in vivo, the biodistribution of samples was observed by IVIS; Lipo2000@siRNA was used as control. After I.V. injection of GNS-P@siRNA-Cy5 to Panc02 tumor-bearing mice for 0 h or 12 h, the biodistribution was observed for fluorescence imaging. As shown in Figure 11a, compared with the fluorescence intensity of Lipo2000@siRNA-Cy5, that of GNS-P@siRNA-Cy5 is almost concentrated in the tumor site. To further visualize the distribution of GNS-P@siRNA in tumors and organs, we euthanized the mice and the tumor and organs were stripped and assessed. As shown in Figure 11b, the fluorescence mainly accumulated in tumors, illustrating the excellent targeting performance of GNS-P@siRNA-Cy5 in vivo. The quantitative analysis of fluorescence intensity, which is consistent with the biodistribution imaging, was shown in Figure 11c. These results were ascribed to the fact that modification of PEG could prolong the circulation time and functionalization of tumor-targeting peptide and could combine to the overexpressed receptor of tumor cells specifically, which leads to significantly amplified tumor accumulation of the nanoplatform GNS-P.

## 4. Conclusions

In this paper, we customized a versatile nanoplatform, GNS-P, that incorporates a prominent siRNA condensing capability, a remarkable tumor-targeting ability and excellent cell internalization efficiency for novel siRNA delivery. Due to the superadditive functionality of the nanoplatform, siRNA could be delivered to the tumor site and be satisfactorily internalized by tumor cells, while the gene therapeutic effect could be remarkably augmented. The in vitro and in vivo results verified the good biocompatibility, low toxicity and extraordinary tumor accumulation capability of GNS-P and indicated that GNS-P could be used as an excellent gene carrier for tumor therapy. This study discusses the basic performance study of GNS-P as a gene carrier, followed by further investigation on the in vivo metabolism level of GNS-P. Moreover, since GNS-P is endowed with an excellent energy conversion ability and photoacoustic/photothermal imaging capability, the nanoplatform could be further used for diagnosis and treatment integration as an efficient anticancer gene therapy. 

## Figures and Tables

**Table 1:** ESI-MS analysis of tumor-targeting peptide.

Peptide	M (Calculated)	*m*/*z* (Found)
CRRRRRRGD	1228.69	[M+H]^+^: 1229.0, 1230.2,1231.2 [M+CF_3_CO+H]^+^: 1326.9, 1328 M+Na]^5+^: 249.1

**Table 2:** Characterization of GNS based nanoplatforms before and after modification.

Sample	Hydrodynamic Diameter (nm)	Zeta Potential (mV)	Longitudinal SPR Peak (nm)
GNS	95.8	−18	724
GNS-PEG	168.4	−13	752
GNS-P	170.2	+14	781

## Data Availability

The data presented in this study are available in this study. Additional information could be available on request from the corresponding author.

## Abbreviations

mRNA, messenger RNA; RNAi, RNA interference; siRNA, small interfering RNA; GNS, gold nanostars; GNS-PEG, PEG modified gold nanostars; GNS-P, cationic tumor-targeting peptide-modified gold nanostars; Lipo2000, Lipofectamine 2000; HPLC, high performance liquid chromatography; ESI-MS, electrospray ionization mass spectrometry; TEM, Transmission Electron Microscopy; SEM, Scanning Electron Miscopy; CLSM, confocal laser scanning microscope; ELISA, enzyme-linked immunosorbnent assay; IVIS, In Vivo Imaging System; GNS @siRNA, siRNA loaded on GNS; GNS-PEG@siRNA, siRNA loaded on GNS-PEG; GNS-P@siRNA, siRNA loaded on GNS-P; Lipo2000@siRNA, siRNA loaded on Lipo2000.

## References

[1] Lu J., Lu G.L., Tan S.D., Xia J., Xiong H.L., Yu X.F., Qi Q.Q., Yu X., Li L., Yu H. (2020). A COVID-19 mRNA vaccine encoding SARS-CoV-2 virus-like particles induces a strong antiviral-like immune response in mice. Cell Res..

[2] Polack F.P., Thomas S.J., Kitchin N., Absalon J., Gurtman A., Lockhart S., Perez J.L., Pérez M.G., Moreira E.D., Zerbini C. (2020). Safety and efficacy of the BNT162b2 mRNA Covid-19 Vaccine. N. Engl. J. Med..

[3] Holjencin C.E., Feinberg C.R., Hedrick T., Halsey G., Williams R.D., Patel P.V., Biles E., Cummings J.C., Wagner C., Vyavahare N. (2021). Advancing peptide siRNA-carrier designs through L/D-amino acid stereochemical modifications to enhance gene silencing. Mol. Ther. Nucleic Acids.

[4] Singh A., Trivedi P., Jain N.K. (2018). Advances in siRNA delivery in cancer therapy. Artif. Cells Nanomed. Biotechnol..

[5] Gavrilov K., Saltzman W.M. (2012). Therapeutic siRNA: Principles, challenges, and strategies. Yale J. Biol. Med..

[6] Rao D.D., Vorhies J.S., Senzer N., Nemunaitis J. (2009). siRNA vs. shRNA: Similarities and differences. Adv. Drug Deliv. Rev..

[7] Ngamcherdtrakul W., Yantasee W. (2019). siRNA therapeutics for breast cancer: Recent efforts in targeting metastasis, drug resistance, and immune evasion. Transl. Res..

[8] Zhang P., An K., Duan X., Xu H., Li F., Xu F. (2018). Recent advances in siRNA delivery for cancer therapy using smart nanocarriers. Drug Discov. Today.

[9] Yang H., Cui W., Wang L. (2019). Epigenetic synthetic lethality approaches in cancer therapy. Clin. Epigenetics.

[10] Jain S., Pathak K., Vaidya A. (2018). Molecular therapy using siRNA: Recent trends and advances of multi target inhibition of cancer growth. Int. J. Biol. Macromol..

[11] Subhan M.A., Torchilin V.P. (2019). Efficient nanocarriers of siRNA therapeutics for cancer treatment. Transl. Res..

[12] Mishra D.K., Balekar N., Mishra P.K. (2017). Nanoengineered strategies for siRNA delivery: From target assessment to cancer therapeutic efficacy. Drug Deliv. Transl. Res..

[13] Kim Y.-D., Park T.-E., Singh B., Maharjan S., Choi Y.-J., Choung P.-H., Arote R.B., Cho C.-S. (2015). Nanoparticle-mediated delivery of siRNA for effective lung cancer therapy. Nanomedicine.

[14] Fakhr E., Zare F., Teimoori-Toolabi L. (2016). Precise and efficient siRNA design: A key point in competent gene silencing. Cancer Gene Ther..

[15] Simonenko V., Lu X., Roesch E., Mutisya D., Shao C., Sun Q., Patterson-Orazem A., McNair M., Shanmuganathan A., Lu P. (2020). A novel siRNA-gemcitabine construct as a potential therapeutic for treatment of pancreatic cancer. NAR Cancer.

[16] Abdelaal A.M., Kasinski A.L. (2021). Ligand-mediated delivery of RNAi-based therapeutics for the treatment of oncological diseases. NAR Cancer.

[17] Mousa J., Chen P. (2009). Peptide mediated siRNA delivery. Curr. Top. Med. Chem..

[18] Yu Z., Zhang X., Pei X., Cao W., Ye J., Wang J., Sun L., Yu F., Wang J., Li N. (2021). Antibody-siRNA conjugates (ARCs) using multifunctional peptide as a tumor enzyme cleavable linker mediated effective intracellular delivery of siRNA. Int. J. Pharm..

[19] Cummings J.C., Zhang H., Jakymiw A. (2019). Peptide carriers to the rescue: Overcoming the barriers to siRNA delivery for cancer treatment. Transl. Res..

[20] Zhu Y., Meng Y., Zhao Y., Zhu J., Xu H., Zhang E., Shi L., Du L., Liu G., Zhang C. (2019). Toxicological exploration of peptide-based cationic liposomes in siRNA delivery. Colloids Surf. B Biointerfaces.

[21] Beloor J., Zeller S., Choi C.S., Lee S.-K., Kumar P. (2015). Cationic cell-penetrating peptides as vehicles for siRNA delivery. Ther. Deliv..

[22] Sindhwani S., Syed A.M., Ngai J., Kingston B.R., Maiorino L., Rothschild J., MacMillan P., Zhang Y., Rajesh N.U., Hoang T. (2020). The entry of nanoparticles into solid tumours. Nat. Mater..

[23] Luther D.C., Huang R., Jeon T., Zhang X., Lee Y.-W., Nagaraj H., Rotello V.M. (2020). Delivery of drugs, proteins, and nucleic acids using inorganic nanoparticles. Adv. Drug Deliv. Rev..

[24] Jiang Z., Thayumanavan S. (2020). Non-cationic material design for nucleic acid delivery. Adv. Ther..

[25] Vaughan H.J., Green J.J., Tzeng S.Y. (2020). Cancer-targeting nanoparticles for combinatorial nucleic acid delivery. Adv. Mater..

[26] Chen S., Fan J.X., Qiu W.X., Liu L.H., Cheng H., Liu F., Yan G.P., Zhang X.Z. (2017). Self-assembly drug delivery system based on programmable dendritic peptide applied in multidrug resistance tumor therapy. Macromol. Rapid Commun..

[27] Wang H., Zhou J., Fu Y., Zheng Y., Shen W., Zhou J., Yin T. (2021). Deeply Infiltrating iRGD-graphene oxide for the geting-based antimigration. Adv. Healthc. Mater..

[28] Chen S., Fan J.X., Liu X.H., Zhang M.K., Liu F., Zeng X., Yan G.P., Zhang X.Z. (2019). A self-delivery system based on an amphiphilic proapoptotic peptide for tumor targeting therapy. J. Mater. Chem. B.

[29] Liu C., Chen Y.X., Wang J.X., Luo X., Huang Y.D., Xu J.L., Yan G.P., Chen S., Zhang X.Z. (2018). A multi-functional drug delivery system based on dendritic peptide for tumor nuclear accurate targeting therapy. Acta Polym. Sin..

[30] Chen S., Lei Q., Qiu W.X., Liu L.H., Zheng D.W., Fan J.X., Rong L., Sun Y.X., Zhang X.Z. (2017). Mitochondria-targeting “Nanoheater” for enhanced photothermal/chemo-therapy. Biomaterials.

[31] Chen S., Fan J., Qiu W., Liu F., Yan G., Zeng X., Zhang X. (2018). A cellular/intranuclear dual-targeting nanoplatform based on gold nanostar for accurate tumor photothermal therapy. J. Mater. Chem. B.

[32] Chen S., Lei Q., Li S.Y., Qin S.Y., Jia H.Z., Cheng Y.J., Zhang X.Z. (2016). Fabrication of dual responsive co-delivery system based on three-armed peptides for tumor therapy. Biomaterials.

[33] Chen S., Fan J.X., Zheng D.W., Liu F., Zeng X., Yan G.P., Zhang X.Z. (2020). A multi-functional drug delivery system based on polyphenols for efficient tumor inhibition and metastasis prevention. Biomater. Sci..

[34] He C., Yu L., Ding L., Chen Y., Hao Y. (2020). Self-assembled/drug-composed nanomedicine for synergistic photonic hyperthermia and targeted therapy of breast cancer by inhibiting ERK, AKT, and STAT3 signaling cascades. Adv. Funct. Mater..

[35] Wang C., Wu B., Wu Y., Song X., Zhang S., Liu Z. (2020). Camouflaging nanoparticles with brain metastatic tumor cell membranes: A new strategy to traverse blood–brain barrier for imaging and therapy of brain tumors. Adv. Funct. Mater..

[36] Fan J.X., Peng M.Y., Wang H., Zheng H.R., Liu Z.L., Li C.X., Wang X.N., Liu X.H., Cheng S.X., Zhang X.Z. (2019). Engineered bacterial bioreactor for tumor therapy via fenton-like reaction with localized H_2_O_2_ generation. Adv. Mater..

[37] Fan J.X., Zheng D.W., Mei W.W., Chen S., Chen S.Y., Cheng S.X., Zhang X.Z. (2017). A metal-polyphenol network coated nanotheranostic system for metastatic tumor treatments. Small.

